# Predictors of Loneliness, Mental Wellbeing, and Stress During the COVID-19 Pandemic in Switzerland

**DOI:** 10.3389/ijph.2026.1609518

**Published:** 2026-02-27

**Authors:** Christopher Zaiser, Nora M. Laskowski, Roland Müller, Georgios Paslakis

**Affiliations:** 1 University Hospital for Psychosomatic Medicine and Psychotherapy, Ruhr University Bochum, Bochum, Germany; 2 Männer.ch, Competence Centre for Men’s Health, Bern, Switzerland

**Keywords:** COVID-19, loneliness, mental wellbeing, risk factors, stress, anxiety, population survey, Switzerland

## Abstract

**Objectives:**

The COVID-19 pandemic substantially affected population mental health. We examined predictors of increased loneliness, reduced mental wellbeing, and elevated stress in Switzerland.

**Methods:**

Using the 2022 Swiss Health Survey, we ran weighted binary logistic regressions (weights representing 7,182,252 residents aged ≥15 years) to test sociodemographic, psychosocial, behavioral, and pandemic-related predictors of perceived change since before the pandemic; extended models added COVID-19 health variables. Predictors were entered in blocks.

**Results:**

Increased general anxiety since the pandemic’s onset was the strongest predictor across outcomes (OR range 3.25–11.31). Younger age, female gender, migration background, and sexual minority status were associated with higher burden. Living alone and strained family or friendship relationships also predicted greater loneliness, reduced wellbeing, and stress. Economic strain, increased alcohol/tobacco use, and higher workload were further associated with adverse outcomes. In extended models, prolonged COVID-19 symptoms were strongly associated with reduced wellbeing.

**Conclusion:**

Pandemic-related burden reflected an interplay of anxiety, relationship strain, and economic adversity. Public health responses should prioritize early identification of anxiety and targeted support for high-risk groups, alongside measures that strengthen social connectedness and adaptive coping.

## Introduction

The COVID-19 pandemic acted as a major global stressor, disrupting daily life and straining societal and personal resources [1, 2]. Mitigation measures (e.g., lockdowns and physical distancing) and related health and economic uncertainties contributed to elevated psychological stress [3, 4]. Global modelling suggests substantial increases in major depressive disorder and anxiety disorders in 2020 [1].

Among the psychological consequences of COVID-19, a rise in loneliness has been particularly notable [3]. Because social connection was curtailed by stay-at-home orders, several studies observed increases in loneliness as early as 2020 [5, 6]. A meta-analysis found a small but significant rise compared with pre-pandemic levels [7]. This matters because loneliness is linked to poorer mental and physical health and premature mortality [8].

Importantly, the mental health burden of the pandemic has not affected everyone the same but has magnified existing social inequalities, with higher distress reported among women, younger individuals, and socioeconomically disadvantaged groups [9]. Relationship status and quality also mattered: individuals who were single or living alone tended to report worse outcomes, whereas emotionally close ties buffered against distress under restrictions [10–12]. Disparities related to sexual orientation have likewise emerged; sexual and/or gender minority individuals reported higher depression, stress, and loneliness in multiple settings and regions [13–15]. In addition, economic strain, especially job or income loss, was consistently linked to anxiety, depression, and psychological distress and may persist beyond the acute crisis [16–20]. Together, these findings underscore that socially disadvantaged groups have borne a disproportionate mental health burden during COVID-19.

During the COVID-19 pandemic, multiple psychosocial stressors and individual coping behaviors have significantly shaped mental health outcomes [21]. Many households experienced heightened tension as prolonged confinement, home-schooling, and remote work blurred personal boundaries and disrupted routines [22, 23]. Facing chronic uncertainty and fears of infection or loss, some individuals turned to maladaptive strategies such as increased alcohol use or smoking [24, 25]. Large surveys across countries also indicate that low resilience – the capacity to adapt positively and recover from adversity [26] – was common and strongly linked to such unhealthy coping behaviors, including higher alcohol and drug use, poorer diet, and excessive self-isolation [27]. While such behaviors may offer short-term relief from (pandemic-related) stress, they may worsen mental health in the long run. These findings highlight how (indirect) behavioral responses have further undermined wellbeing during the pandemic.

Age and gender were consistently associated with mental health impacts during the pandemic. Across studies, younger people – especially adolescents and young adults – often reported higher levels of distress, loneliness, and stress than older adults, potentially reflecting stronger disruptions to education, social life, and early career prospects [28–33]. Gender differences were also robust, with women reporting higher distress, partly linked to increased caregiving and work–life disruptions [34–38]. Disordered eating and related burdens were reported more frequently among women and individuals from sexual and gender minority groups in some studies [39].

Given this background, there is an urgent need to identify the specific risk factors for increased loneliness, heightened stress, and reduced mental wellbeing during COVID-19. While many studies have explored mental health outcomes during the pandemic, relatively few have used representative population surveys to comprehensively examine demographic, social, and psychosocial predictors in a national context. The present study addresses this gap by analyzing data from a large, representative Swiss population survey conducted during the pandemic in 2022. We investigate a broad range of potential risk factors – including age, gender, relationship status, sexual orientation, financial strain, psychosocial stressors, and coping behaviors – as predictors of three outcomes: retrospectively assessed perceived change since before the pandemic in loneliness, mental wellbeing, and stress. These findings contribute to the broader international literature and provide an evidence base to guide targeted mental health interventions and policy responses for current and future public health crises.

## Methods

### Questionnaire

This study is based on data from the 2022 Swiss Health Survey (BFS [40]), a nationally representative survey conducted every 5 years. The survey collects comprehensive data on health status, behaviors, and determinants of health in the general population. Since 2007, the questionnaire has incorporated elements of the European Health Interview Survey (EHIS), enabling international comparability.

In 2022, as a unique addition to this survey wave, participants retrospectively assessed how their health, psychosocial experience, and behaviors changed since the onset of the COVID-19 pandemic.

#### Perceived Changes in Loneliness, Mental Wellbeing, and Stress

Three core items in the 2022 Swiss Health Survey were used to assess perceived changes in psychological functioning during the COVID-19 pandemic. These assessed perceived change since before the pandemic in (i) loneliness frequency, (ii) overall mental wellbeing, and (iii) perceived stress.

Each item was measured using a five-point Likert scale, capturing the direction and magnitude of perceived change (e.g., “much more often” to “much less often” for loneliness, “much better” to “much worse” for wellbeing, and “much higher” to “much lower” for stress).

#### Additionally Assessed Perceived Changes

Beyond the main outcomes, the survey included a range of items addressing perceived changes due to the pandemic in social relationships, health behaviors, and broader life circumstances. These included:

- *Interpersonal relationships:* with family members and with friends/acquaintances
- *Health-related behaviors:* alcohol consumption, tobacco use, and physical activity
- *Physical and psychological indicators:* body weight and generalized anxiety
- *Economic and occupational conditions:* workload and household income

All variables were measured using five-point Likert scales indicating change (e.g., “much better” to “much worse” or “much higher” to “much lower”), with an additional response option for “not applicable” where appropriate.

#### COVID-19 Symptoms

Participants reported whether they had been infected with COVID-19 and whether they had received a positive Polymerase Chain Reaction (PCR) test. A list of ten common symptoms associated with COVID-19 was presented, including fever, fatigue, respiratory complaints, digestive issues, and loss of taste or smell. Each symptom was recorded as either present or absent (“applies”/“does not apply”). The subjective duration of illness was captured using predefined categories ranging from “a few days to 1 week” to “more than 8 weeks.” Hospitalization due to COVID-19 was assessed, including duration of the hospital stay (if applicable), using the same predefined time categories as for illness duration.

### Sampling and Procedures

The 2022 Swiss Health Survey used a stratified random sampling approach based on cantonal and municipal resident registers, updated quarterly. Cantons served as strata to ensure regional representativeness through minimum sample sizes per region. The target population included all individuals aged 15 and older living in private households in Switzerland, including non-Swiss nationals with valid residence permits. While the national core sample comprised 10,000 individuals, 17 cantons and the city of Zurich opted for sample expansions.

Data collection followed a mixed-methods design. Most participants completed computer-assisted telephone interviews, with computer-assisted personal interviews offered as alternatives. In total, 21,930 telephone interviews were completed, with a response rate of 36.2%. Following the telephone interviews, participants completed a written questionnaire online (74%) or via paper-and-pencil (26%), yielding 19,137 valid responses (90.1% response rate). Interviews were conducted in German, French, and Italian, corresponding to the Swiss language regions, over the course of the calendar year 2022. Quarterly sampling was evenly distributed to account for seasonal variation.

Rigorous quality control procedures ensured data integrity, including real-time plausibility checks, manual validation of inconsistencies, and cross-referencing of demographic data with registry information. All participants provided informed consent prior to participation in the Swiss Health Survey 2022 and participation was voluntary. The survey was conducted by the Swiss Federal Statistical Office (BFS) in accordance with Swiss data protection regulations, and data were anonymized before being made available for secondary analysis.

### Analyses

Only participants who completed both the interview and the written survey were included in the analyses; the respective datasets were merged prior to analysis. Additionally, the data used in this study are owned by the Swiss Federal Statistical Office and cannot be shared publicly due to licensing restrictions.

#### Weighting

The 2022 Swiss Health Survey [40] provides a representative sample of the population aged 15 years and older living in private households in Switzerland. To ensure that the survey data accurately reflect the structure of the Swiss population, weighting procedures were applied. These adjustments account for both the sampling design and relevant demographic characteristics.

The unweighted sample sizes are reported in the Sampling and Procedures section (21,930 completed telephone interviews; 19,137 valid questionnaire responses). All analyses used survey weights, yielding a weighted population total of 7,182,252. The weighting process involved comparing the sample composition with known demographic benchmarks and correcting for potential discrepancies [40].

Regional disproportionalities were adjusted to reflect the actual distribution of the population across cantons. The initial base weights considered the sampling design, including selection probabilities and patterns of non-response. To further address differential non-response, an additional model-based non-response adjustment was applied. This model incorporated response behavior patterns based on key characteristics.

Finally, a calibration weighting step ensured that the final sample aligned with known population distributions for variables such as household size, gender, nationality, marital status, and age group. The most important factors considered in the weighting procedure included region of residence, gender, age, nationality, marital status, and household size. This process ensured that the weighted dataset accurately reflected the demographic structure of the Swiss population. All analyses were conducted using weighted data.

#### Statistical Analysis

**FIGURE 1:**
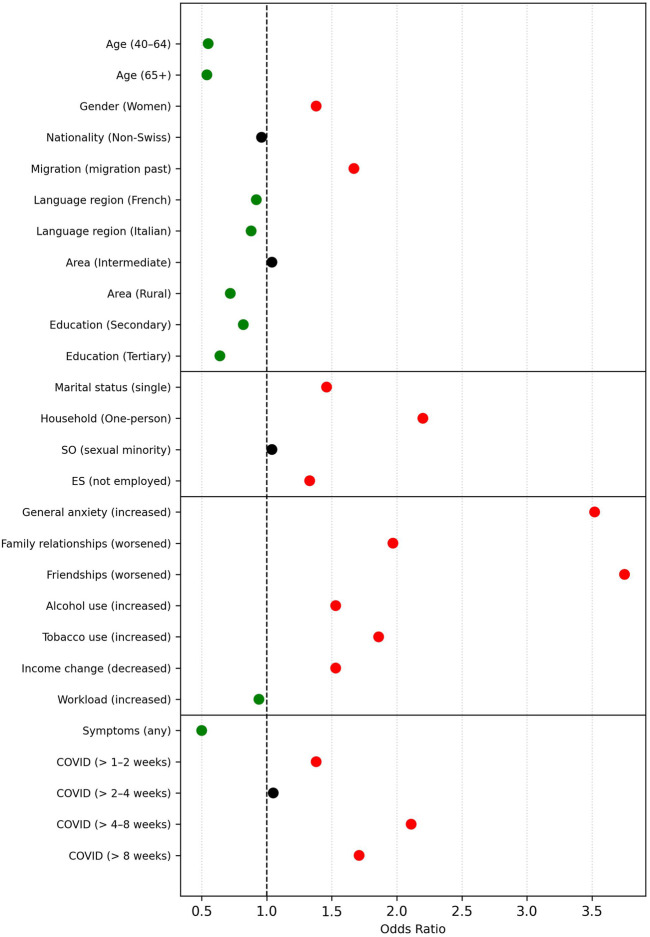
Risk and Protective Factors for Pandemic-Related Loneliness (Swiss Health Survey, Switzerland, 2022). *Note.* SO = sexual orientation; ES = employment status. Predictors are listed with the respective comparison group in parentheses (e.g., *Gender (Women)* compares women to the reference group: men). Green dots indicate protective factors (odds ratio <0.95), red dots represent risk factors (odds ratio >1.05). Predictors with odds ratios between 0.95 and 1.05 are marked in black, indicating negligible practical relevance.

**FIGURE 2:**
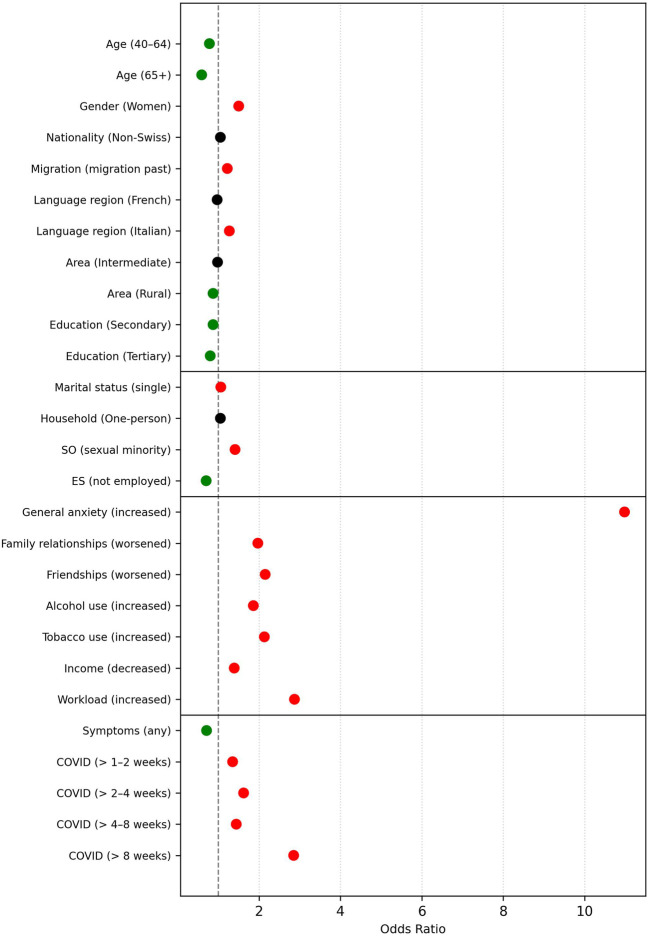
Risk and Protective Factors for Decreased Mental Wellbeing: Extended Logistic Regression Results (Swiss Health Survey, Switzerland, 2022). *Note.* SO = sexual orientation; ES = employment status. Predictors are listed with the respective comparison group in parentheses (e.g., *Gender (Women)* compares women to the reference group: men). Green dots indicate protective factors (odds ratio <0.95), red dots represent risk factors (odds ratio >1.05). Predictors with odds ratios between 0.95 and 1.05 are marked in black, indicating negligible practical relevance.

**FIGURE 3:**
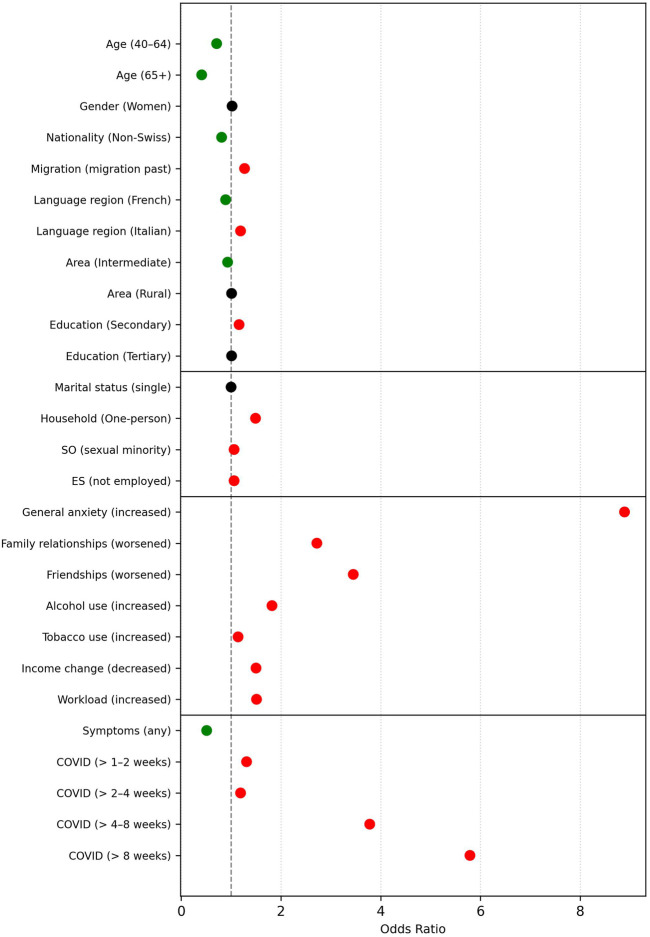
Risk and Protective Factors for Increased Stress During the Pandemic: Visual Summary of the Logistic Regression Model (Swiss Health Survey, Switzerland, 2022). *Note.* SO = sexual orientation; ES = employment status. Predictors are listed with the respective comparison group in parentheses [e.g., *Gender (Women)* compares women to the reference group: men]. Green dots indicate protective factors (odds ratio <0.95), red dots represent risk factors (odds ratio >1.05). Predictors with odds ratios between 0.95 and 1.05 are marked in black, indicating negligible practical relevance.

All statistical analyses were conducted using IBM SPSS Statistics, version 30.0.0.0 [41]. Figures 1–3 were created using Python version 3.12.4 [42] for Windows in a Jupyter Notebook environment [43], employing the matplotlib (v3.9.0 [44]) and pandas (v2.2.2 [45]) libraries. Descriptive statistics were reported as absolute (n) and relative frequencies (%), or as means (*M*) and standard deviations (*SD*) for continuous variables.

To examine predictors of psychological burden during the COVID-19 pandemic, separate binary logistic regression models were estimated for each of the three outcome variables. All outcomes were dichotomized based on participants’ responses as follows:

- *Increased loneliness*: responses of “much more often” or “more often” were coded as 1
- *Reduced mental wellbeing*: responses of “worse” or “much worse” were coded as 1
- *Increased perceived stress*: responses of “higher” or “much higher” were coded as 1

All other responses were coded as 0 (no deterioration or improvement). Predictor variables were likewise dichotomized to reflect negative developments (e.g., “increased alcohol use”) where theoretically meaningful. Categorical variables with more than two levels (e.g., age, language region) were dummy-coded, using the first category as reference (see Table 1). Predictors were entered in thematic blocks to assess their incremental changes in model fit (Nagelkerke *R* ^2^). Nagelkerke’s R^2^ was used as a descriptive pseudo-R^2^ index of model fit and is not interpreted as variance explained. Two model variants were estimated for each outcome:

- A *core model* (sociodemographic, social/psychosocial factors), maximizing sample size (88.4%–88.8%)
- An *extended model* including COVID-19-specific predictors, with a reduced analytic sample (43.9%–44.0%) due to missing data

**TABLE 1:** Overview of predictor variables and coding used in logistic regression models (Swiss Health Survey, Switzerland, 2022).

Thematic block	Variable	Categories^a^
Sociodemographic	Age (categorial)	15–39; 40–64; 65+
	Gender	Male; female
	Nationality	Swiss; non-swiss
	Migration background	None; first generation or higher
	Language region	German, French, Italian
	Residential area	Urban; intermediate; rural
	Education	Compulsory; secondary; tertiary
Social	Marital status	With partner^b^; single^c^
	Household type	Multi-person household; one-person household
	Sexual orientation	Heterosexual; sexual minority
	Employment status	Employed; not employed
Psychosocial stressors	Increased general anxiety	No; yes
	Worsened family relationship	No; yes
	Worsened friendships	No; yes
	Increased alcohol use	No; yes
	Increased tobacco use	No; yes
	Loss of income	No; yes
	Increased workload	No; yes
COVID-19-related^d^	Reported COVID-19 symptoms	None; any reported
	Duration COVID-19 illness	≤1 week; >1–2 weeks; >2–4 weeks; >4–8 weeks; >8 weeks

^a^ The first listed category was used as the reference category in all regression models.

^b^ With partner = married or in a registered partnership.

^c^ Single = single, widowed, divorced, unmarried, or in a dissolved registered partnership.

^d^ COVID-19-related predictors were only included in a separate extended model due to substantial missing data.

Missing data were handled using complete-case analysis within each model. Net household income was excluded from the regression models due to substantial item non-response (59.8%), which would have led to considerable case loss in the core analyses; to still capture financial strain, we retained perceived income loss since the onset of the pandemic as an alternative indicator with markedly lower missingness. In contrast, COVID-19 illness variables were included in a separate extended model because they address a distinct, pandemic-specific research question (i.e., whether illness experiences add explanatory value beyond sociodemographic, social, psychosocial, and behavioral predictors). Given the substantial missingness and the resulting reduction in analytic sample size, extended models are presented as exploratory and should be interpreted as conditional on the reduced subsample with complete COVID-19 information, while the core models represent the primary results.

Table 1 provides an overview of all predictors included, their coding, and reference categories. Odds ratios (ORs) with 95% confidence intervals (CIs) were reported for each model, along with additional statistics (unstandardized regression coefficient [B]; standard error [SE]; Wald statistic; Nagelkerke R^2^) to assess the strength, direction, and significance of associations.

## Results

### Sample Characteristics

The final sample consisted of 7,182,252 weighted cases. The mean age of included cases was 48.9 years (*SD* = 19.0), with a range from 15 to 100 years. In terms of gender identity, the weighted sample included 49.1% cisgender men and 50.0% cisgender women. Additionally, 0.2% identified as transgender men, 0.3% as transgender women, and a small proportion (<0.5%) as “other” genders (i.e., nonbinary or “other” without specification), with some missing responses (1.3%). The distribution of sexual orientation showed that 92.9% identified as heterosexual, while 2.0% reported a homosexual orientation, 2.3% bisexual, and 2.8% “other” and/or not specified.

In terms of education, 40.2% held a tertiary degree. A quarter of cases (24.6%) were non-Swiss nationals, 29.6% had a first-generation migration background, and 7.8% belonged to the second or higher generation. Urban areas accounted for 62.0% of the population, and most respondents lived in the German-speaking region (71.6%).

Employment status indicated that 67.1% were employed, 2.1% were unemployed but generally able to work, and 30.8% were not part of the labor force (e.g., due to retirement, disability, caregiving responsibilities, or military service). Because of a high rate of missing responses (59.8%), income data were excluded from the regression models to avoid substantial case loss. Descriptive details for all variables, including the three main pandemic-related outcome variables examined in the regression analyses (loneliness, wellbeing, stress), are presented in Table 2.

**TABLE 2:** Weighted sample characteristics (Swiss Health Survey, Switzerland, 2022).

Variable	Weighted estimate (n (%) or Mean (SD); median (range))
Age	48.92 (18.96); 48.00 (15–100)
Age group	*n* (%)
15–39	2,505,533 (34.9)
40–64	3,040,251 (42.3)
65+	1,636,467 (22.8)
Gender identity (missing cases 92,768 [1.3%])	*n* (%)
Cisgender men	3,481,960 (49.1)
Cisgender women	3,545,610 (50.0)
Trans men	17,448 (0.2)
Trans women	21,691 (0.3)
Men with “other”^a^ gender identity	6,271 (0.1)
Women with “other”^a^ gender identity	16,504 (0.2)
Sexual orientation (missing cases 480,321 [6.7%])	*n* (%)
Heterosexual	6,227,732 (92.9)
Homosexual	134,330 (2.0)
Bisexual	151,061 (2.3)
“Other”, not specified	188,809 (2.8)
Residential area	*n* (%)
Urban	4,455,742 (62.0)
Intermediate (dense peri-urban and rural areas)	1,557,948 (21.7)
Rural	1,168,562 (16.3)
Language region	*n* (%)
German-speaking	5,144,800 (71.6)
French-speaking	1,724,763 (24.0)
Italian-speaking	312,689 (4.4)
Household type (missing cases 5,706 [0.1%])	*n* (%)
Couple with child(ren)	2,759,089 (38.4)
Couple without child(ren)	2,313,034 (32.2)
One-person	1,437,941 (20.0)
Non-family multi-person	267,197 (3.7)
Multi-family	31,960 (0.4)
Single parent	367,325 (5.1)
Marital status	*n* (%)
Single^b^	3,643,026 (50.7)
With partner^c^	3,539,226 (49.3)
Employment status (missing cases 1,047 [<0.1%])	*n* (%)
Employed	4,820,440 (67.1)
Unemployed	151,205 (2.1)
Non-employed	2,209,560 (30.8)
Net monthly income^d^ (missing cases 4,294,405 [59.8%])	Mean (SD); median (range)
​	8,238.50 (14,932.75); 8,238.50 (0–1,000,000)
Income^d^ group (missing cases 4,294,405 [59.8%])	*n* (%)
≤4,000	487,889 (36.7)
>4,000–8,000	520,387 (39.2)
>8,000	319,582 (24.1)
Education (missing cases 49,854 [0.7%])	*n* (%)
Compulsory education	1,071,035 (15.0)
Upper secondary education	3,193,274 (44.8)
Tertiary education	2,868,090 (40.2)
Migration background (missing cases 58,067 [0.8%])	*n* (%)
None	4,463,392 (62.7)
First generation	2,107,469 (29.6)
≥ Second generation	553,324 (7.8)
Nationality	*n* (%)
Swiss	5,418,020 (75.4)
Non-swiss	1,764,232 (24.6)
Pandemic-related outcomes^e^	*n* (%)
Perceived increase in loneliness	675,825 (9.4)
Perceived reduction in mental wellbeing	928,217 (12.9)
Perceived increase in stress	1,448,827 (20.2)

^a^ “Other” gender identity = cases coded as non-binary and/or indicating a gender identity not listed among the predefined options without specification.

^b^ Single = single, widowed, divorced, unmarried, or in a dissolved registered partnership.

^c^ With partner = married or in a registered partnership.

^d^ Values refer to net monthly household income in Swiss francs.

^e^ Pandemic-related outcome variables represent dichotomous indicators reflecting perceived deterioration compared to the pre-pandemic period.

### Regression Models Predicting Pandemic-Related Outcomes

To examine the predictors of retrospectively assessed perceived deterioration since before the pandemic in loneliness, reduced mental wellbeing, and increased stress during the COVID-19 pandemic, separate binary logistic regression models were estimated for each outcome. All models included sociodemographic characteristics, social factors, and psychosocial or behavioral stressors (core models). COVID-specific variables were added in extended models. Outcomes were dichotomized (see section *Statistical Analysis*).

All three outcomes reflect self-reported perceived change compared with the pre-pandemic period, assessed at a single time point in 2022.

#### Increased Loneliness

Initial analyses based on 88.4% of the weighted sample demonstrated acceptable model fit (Nagelkerke’s *R* ^2^ = 0.212), *χ* ^2^ [22] = 655,757.31, *p* < 0.001. Older age was linked to lower odds of increased loneliness (OR = 0.58 for individuals aged 40–64; OR = 0.57 for those aged 65+, compared to the 15–39 reference group). Higher odds were observed for women (OR = 1.32), individuals with a migration background (OR = 1.60), and those living alone (OR = 1.96). Psychosocial stressors showed particularly strong associations: worsened friendships (OR = 3.48), increased anxiety (OR = 3.25), and strained family relationships (OR = 1.81) were all significant predictors. Behavioral and economic factors such as increased alcohol use (OR = 1.50), tobacco use (OR = 1.77), and income deterioration (OR = 1.46) were also linked to greater loneliness.

When COVID-19-specific variables were included in the extended model (43.9% of the weighted sample), model fit improved (Nagelkerke’s *R* ^2^ = 0.246), *χ* ^2^ [27] = 391,768.10, *p* < 0.001. Most predictors remained significant, with comparable effect sizes. Notably, illness duration played a role: compared to those whose symptoms lasted only a few days to 1 week, individuals with symptoms lasting 2–4 weeks had more than twice the odds of increased loneliness (OR = 2.11), and those with symptoms beyond 8 weeks also showed elevated odds (OR = 1.71). Interestingly, merely having had COVID-19 symptoms – regardless of duration – was associated with lower odds of loneliness (OR = 0.50). Figure 1 visualizes the results, highlighting protective and risk factors for each outcome, while detailed model estimates are provided in Supplementary Table S1.

#### Reduced Psychological Wellbeing

The core model for reduced mental wellbeing included 88.8% of the weighted sample and showed good model fit (Nagelkerke’s *R* ^2^ = 0.324), *χ* ^2^ [22] = 1,234,377.36, *p* < 0.001. Older individuals reported lower odds of wellbeing deterioration (e.g., OR = 0.45 for age 65+ vs. 15–39). Female gender (OR = 1.10), migration background (OR = 1.24), and living alone (OR = 1.32) were each linked to higher risk. Strongest associations emerged for increased anxiety (OR = 8.57), worsened friendships (OR = 3.39), family strain (OR = 2.70), and increased alcohol use (OR = 2.04). Smoking (OR = 1.64), income loss (OR = 1.43), and increased workload (OR = 1.49) were also relevant.

Incorporating COVID-19 illness variables in the extended model (based on 44.0% of the sample) yielded a slightly improved model fit (Nagelkerke’s *R* ^2^ = 0.336), *χ* ^2^ [27] = 664,795.00, *p* < 0.001. The duration of illness emerged as a strong factor: participants whose symptoms lasted two to 4 weeks had over three times the odds of reduced wellbeing (OR = 3.78), while symptoms persisting beyond 8 weeks were associated with nearly sixfold higher odds (OR = 5.79), compared to the reference group (symptoms lasting only a few days to 1 week). Like loneliness, reporting any COVID-19 symptoms at all was linked to slightly lower odds of deterioration (OR = 0.51). Figure 2 illustrates the adjusted odds ratios for reduced mental wellbeing based on the regression model; full results are presented in Supplementary Table S2.

#### Increased Stress

In the model predicting increased perceived stress, 88.7% of the weighted sample was retained. Model fit was strong (Nagelkerke’s R^2^ = 0.343), χ^2^ [22] = 1,578,587.60, p < 0.001. Lower odds of stress were observed for older individuals (e.g., OR = 0.55 for age 65+). In contrast, women (OR = 1.47) and those with a migration background (OR = 1.23) showed elevated stress levels. The most pronounced associations were found for worsened anxiety (OR = 11.31), strained family and friendship relationships (ORs = 2.06), increased alcohol use (OR = 1.80), tobacco use (OR = 2.14), and income loss (OR = 1.46). Work-related deterioration, particularly increased workload, was a strong contributor (OR = 2.70).

Further analyses incorporating COVID-19 illness duration (44.0% of the sample) led to modest gains in model fit (Nagelkerke’s *R* ^2^ = 0.352), *χ* ^2^ [27] = 839,631.66, *p* < 0.001. Symptom duration had a clear effect: individuals ill for more than 8 weeks had nearly threefold higher odds of stress (OR = 2.85). Elevated odds were also seen for symptom durations of 2–4 weeks (OR = 1.62) and 4–8 weeks (OR = 1.44), relative to the reference group (few days to 1 week). Figure 3 provides a visual representation of the adjusted odds ratios for increased stress, while the full model results are available in Supplementary Table S3.

## Discussion

This study identified key sociodemographic, social, psychosocial, and pandemic-specific predictors of retrospectively assessed perceived deterioration since before the pandemic in loneliness, mental wellbeing, and stress in Switzerland. Drawing on a large, representative sample, the findings align with prior international research and extend current knowledge by quantifying pandemic-related psychological burden across diverse population groups in a national context.

Among all predictors examined, increased general anxiety stood out as the strongest and most robust factor. Individuals reporting heightened anxiety since the onset of the pandemic had substantially higher odds of increased loneliness, reduced wellbeing, and elevated stress, even after adjustment for sociodemographic, social, and behavioral variables. This underscores the central role of anxiety as a transdiagnostic indicator of pandemic-related distress and is consistent with international evidence linking anxiety symptoms to health fears, economic insecurity, social isolation, and chronic uncertainty [1, 4]. Moreover, the strong association between anxiety and all three outcomes suggests that anxiety may represent a key marker of pandemic-related burden and may co-occur with other adversities such as social strain or financial loss [21, 27]. Potential mediating pathways should be examined in longitudinal research.

Sociodemographic differences revealed additional shared predictors across outcomes. Younger individuals reported higher vulnerability, echoing previous findings that younger age groups, contrary to early expectations, experienced heightened psychological distress during the pandemic [3, 28, 29, 31]. Older age was associated with lower odds of reduced wellbeing and stress, suggesting greater resilience among older adults – potentially due to more stable life circumstances, including financial security through retirement, fewer work-related uncertainties (e.g., job loss or childcare), and better access to support structures (e.g., community or home care services), as well as more advanced emotional regulation [32].

Gender disparities were also evident, with women showing consistently higher odds of increased stress, and moderately elevated odds for loneliness and reduced wellbeing. This aligns with previous research attributing greater psychological tolls among women to increased caregiving burdens and work-life disruptions [34, 35, 38, 39]. Similarly, individuals with a migration background and non-heterosexual orientation faced elevated risks of psychological burden, reflecting structural inequalities, minority stress mechanisms, and heightened social isolation during the pandemic [9, 13, 14].

Social and relational factors played a prominent role across all outcomes. Living alone and being single were strongly associated with loneliness and, to a lesser extent, with reduced wellbeing and stress. These findings reinforce the protective role of close social ties and intimate relationships in buffering against pandemic-related isolation and stress [10, 11]. Crucially, individuals with worsened family or friendship relationships exhibited among the highest odds of adverse psychological outcomes. This highlights the importance of relational stability in times of crisis and is consistent with prior research suggesting that emotionally close ties may be associated with lower psychological burden, especially for adolescents and young adults [12].

Behavioral stressors also showed cross-cutting relevance. Increased alcohol and tobacco use were consistently associated with higher levels of loneliness, stress, and reduced wellbeing, suggesting suggesting that increased substance use may be used as a coping response and is associated with higher psychological burden [24, 25, 27]; however, directionality cannot be determined from the present data. Similarly, income loss and increased workload emerged as strong predictors of mental distress, reflecting the significant mental health impact of economic insecurity and work-related strain, as reported in previous research [16, 17, 20]. These findings illustrate that, in the context of a global and multifaceted crisis, many individuals resorted to short-term maladaptive coping strategies such as increased substance use. This underlines the need for preventive mental health measures that address such behaviors early and promote healthier coping options during future societal disruptions.

Pandemic-specific health factors also added explanatory value in extended models. Prolonged COVID-19 symptoms (especially >8 weeks) were significantly associated with reduced wellbeing and increased stress, while shorter symptom durations showed more modest associations. This finding supports prior evidence on the psychological toll of prolonged illness, including post-acute COVID-19 symptoms [1]. Interestingly, reporting any symptoms *per se* (regardless of duration) was associated with slightly lower odds of deterioration in loneliness and wellbeing. In addition to perceived immunity, this association could be due to unmeasured factors (e.g., temporary changes in responsibilities or increased support during illness), which warrants further investigation.

Taken together, the findings suggest that psychological burden during the pandemic was shaped by an interplay of structural vulnerabilities (e.g., age, gender, migration status), social connectedness, coping behaviors, and illness-related experiences. The recurrence of key predictors, particularly general anxiety, relationship strain, and behavioral and economic stressors, across all three outcomes points to shared mechanisms of psychological vulnerability. These insights highlight the importance of integrated mental health strategies that address both individual vulnerabilities and structural determinants, to ensure timely support and better protection of at-risk populations in the face of future societal disruptions.

### Limitations and Future Directions

While this study provides valuable insights into mental health dynamics during the pandemic, several limitations should be acknowledged to contextualize the findings. First, the cross-sectional design precludes causal inference, and retrospective change ratings may be vulnerable to recall bias. Second, all measures were self-reported and the single-item, non-validated outcome measures may reduce measurement precision. Because the outcomes were measured using single-item indicators of perceived change, direct comparability with studies relying on validated multi-item scales is limited. This may have reduced measurement precision and could attenuate associations; therefore, results should be interpreted primarily in terms of patterns of association rather than scale-equivalent effect sizes. Third, household income had substantial missingness (nearly 60%) and was excluded from regression models, limiting conclusions about absolute financial resources as a determinant of mental health. Because income is a sensitive survey topic, some respondents may have skipped this item, which may introduce non-response bias. Therefore, conclusions regarding absolute financial resources should be interpreted cautiously; we included perceived income loss as an indicator of pandemic-related financial strain. This limits conclusions, given that economic strain was a consistent predictor in other domains and has been widely shown to affect psychological outcomes during the pandemic [18, 19]. Fourth, dichotomizing predictors and outcomes, while pragmatic for logistic regression, likely reduced sensitivity to variation and may have obscured more nuanced patterns. Moreover, important psychosocial constructs such as coping strategies, perceived social support, or caregiving burdens were not assessed, despite their demonstrated relevance in previous pandemic studies [21, 27]. Finally, extended models including COVID-19 illness variables relied on a reduced analytic sample and data were collected at a single time point in 2022, which does not capture within-person trajectories over the pandemic. Because COVID-19 illness variables were only available for a reduced subset and showed substantial missingness, these extended-model findings may be affected by selection and should not be generalized to the full survey population with the same confidence as the core models.

Despite these limitations, the study benefits from a nationally representative dataset and comprehensive predictor modeling, providing a solid basis for identifying key mental health risk factors and social disparities. Future research should prioritize longitudinal designs with validated measures and incorporate qualitative approaches to capture the lived experiences and evolving needs of vulnerable populations. In parallel, establishing ongoing representative monitoring systems that track both risk and protective factors (e.g., resilience, coping strategies, digital connectedness) could improve preparedness and enable timelier, evidence-based responses when large-scale disruptions occur.

This study provides compelling evidence that the psychological burden of the COVID-19 pandemic in Switzerland was not evenly distributed, but strongly shaped by sociodemographic background, relational dynamics, maladaptive behaviors, and subjective health experiences. Younger age, female gender, single status, minority background, worsened relationships, and behavioral or economic stressors were all associated with greater odds of loneliness, reduced wellbeing, and stress. The findings underline the importance of multidimensional, equity-informed public health responses that address social vulnerability, strengthen interpersonal networks, and promote adaptive coping across populations. Tailored interventions targeting high-risk groups and fostering psychosocial resilience are essential for mitigating long-term mental health consequences – both in the aftermath of COVID-19 and in future public health crises.
