# Supplementary material for: Predictors of Loneliness, Mental Wellbeing, and Stress During the COVID-19 Pandemic in Switzerland
Source: Int J Public Health. 2026 Feb 27;71:1609518. doi: 10.3389/ijph.2026.1609518 (PMC12982157; doi:10.3389/ijph.2026.1609518)
Supplement: Supplementary file 1 [file Table1.docx]

**Supplementary Table 1.**

*Binary Logistic Regression Predicting Increased Loneliness.*

| Predictor (comparison group) | Core Model | | | Extended Model | | |  |
| --- | --- | --- | --- | --- | --- | --- | --- |
|  | *B* | OR | 95% CI | *B* | OR | 95% CI | |
| Age (40 – 64) | -0.55 | 0.58 | 0.574–0.582 | -0.59 | 0.55 | 0.548–0.558 | |
| Age (65+) | -0.56 | 0.57 | 0.563–0.575 | -0.61 | 0.54 | 0.535–0.554 | |
| Gender (Women) | 0.27 | 1.32 | 1.308–1.323 | 0.32 | 1.38 | 1.365–1.388 | |
| Nationality (Non-Swiss) | 0.11 | 1.11 | 1.104–1.124 | -0.04 | 0.96 | 0.945–0.969 | |
| Migration (migration past) | 0.47 | 1.60 | 1.586–1.613 | 0.51 | 1.67 | 1.646–1.684 | |
| Language region (French) | 0.03 | 1.03 | 1.020–1.034 | -0.08 | 0.92 | 0.915–0.933 | |
| Language region (Italian) | 0.21 | 1.24 | 1.222–1.254 | -0.13 | 0.88 | 0.862–0.897 | |
| Area (Intermediate) | -0.01 | 0.99 | 0.987–1.001 | 0.04 | 1.04 | 1.032–1.054 | |
| Area (Rural) | -0.37 | 0.69 | 0.682–0.695 | -0.32 | 0.72 | 0.715–0.734 | |
| Education (Secondary) | -0.33 | 0.72 | 0.715–0.727 | -0.19 | 0.82 | 0.814–0.834 | |
| Education (Tertiary) | -0.41 | 0.67 | 0.660–0.672 | -0.45 | 0.64 | 0.633–0.649 | |
| Marital status (single) | 0.25 | 1.29 | 1.279–1.298 | 0.38 | 1.46 | 1.442–1.472 | |
| Household (One-person) | 0.67 | 1.96 | 1.947–1.977 | 0.79 | 2.20 | 2.178–2.226 | |
| SO (sexual minority) | 0.02 | 1.02 | 1.004–1.025 | 0.03 | 1.04 | 1.020–1.050 | |
| ES (not employed) | 0.22 | 1.25 | 1.238–1.258 | 0.29 | 1.33 | 1.319–1.349 | |
| General anxiety (increased) | 1.18 | 3.25 | 3.225–3.268 | 1.26 | 3.52 | 3.488–3.553 | |
| Family relationships (worsened) | 0.60 | 1.81 | 1.796–1.829 | 0.68 | 1.97 | 1.940–1.991 | |
| Friendships (worsened) | 1.25 | 3.48 | 3.451–3.499 | 1.32 | 3.75 | 3.711–3.784 | |
| Alcohol use (increased) | 0.40 | 1.50 | 1.482–1.513 | 0.42 | 1.53 | 1.505–1.547 | |
| Tobacco use (increased) | 0.57 | 1.77 | 1.749–1.791 | 0.62 | 1.86 | 1.827–1.887 | |
| Income (decreased) | 0.38 | 1.46 | 1.453–1.473 | 0.42 | 1.53 | 1.513–1.542 | |
| Workload (increased) | 0.10 | 1.11 | 1.097–1.115 | -0.06 | 0.94 | 0.930–0.952 | |
| Symptoms (any) |  |  |  | -0.69 | 0.50 | 0.487–0.515 | |
| COVID (> 1 – 2 weeks) |  |  |  | 0.32 | 1.38 | 1.366–1.393 | |
| COVID (> 2 – 4 weeks) |  |  |  | 0.05 | 1.05 | 1.030–1.066 | |
| COVID (> 4 – 8 weeks) |  |  |  | 0.75 | 2.11 | 2.054–2.175 | |
| COVID (> 8 weeks) |  |  |  | 0.54 | 1.71 | 1.653–1.764 | |

*Notes*. *B* = logistic regression coefficient; OR = odds ratio; 95% CI = 95% confidence interval for the OR; SO = Sexual orientation; ES = Employment status. Predictors are listed with the respective comparison group in parentheses (e.g., Gender (Women) compares women to the reference group: men). All coefficients were statistically significant at *p* < .001, except for Sexual orientation (homosexual) in the core model (*p* = .005) and Area (Intermediate) (*p* > .05); all standard errors ranged between 0.003 and 0.017.
