# Supplementary material for: Predictors of Loneliness, Mental Wellbeing, and Stress During the COVID-19 Pandemic in Switzerland
Source: Int J Public Health. 2026 Feb 27;71:1609518. doi: 10.3389/ijph.2026.1609518 (PMC12982157; doi:10.3389/ijph.2026.1609518)
Supplement: Supplementary file 2 [file Table2.docx]

**Supplementary Table 2.**

*Binary Logistic Regression Predicting Reduced Psychological Wellbeing.*

| Predictor (comparison group) | Core Model | | | Extended Model | | |  |
| --- | --- | --- | --- | --- | --- | --- | --- |
|  | *B* | OR | 95% CI | *B* | OR | 95% CI | |
| Age (40 – 64) | -0.27 | 0.76 | 0.757–0.767 | -0.35 | 0.71 | 0.702–0.715 | |
| Age (65+) | -0.79 | 0.46 | 0.450–0.460 | -0.90 | 0.41 | 0.402–0.416 | |
| Gender (Women) | 0.10 | 1.10 | 1.096–1.107 | 0.02 | 1.02 | 1.011–1.026 | |
| Nationality (Non-Swiss) | -0.10 | 0.91 | 0.901–0.916 | -0.21 | 0.81 | 0.798–0.817 | |
| Migration (migration past) | 0.21 | 1.24 | 1.229–1.248 | 0.24 | 1.27 | 1.258–1.285 | |
| Language region (French) | 0.04 | 1.04 | 1.029–1.042 | -0.11 | 0.89 | 0.884–0.900 | |
| Language region (Italian) | 0.09 | 1.09 | 1.076–1.104 | 0.17 | 1.19 | 1.170–1.211 | |
| Area (Intermediate) | -0.08 | 0.92 | 0.914–0.927 | -0.07 | 0.93 | 0.921–0.939 | |
| Area (Rural) | -0.15 | 0.86 | 0.852–0.866 | 0.01 | 1.01 | 1.001–1.023 | |
| Education (Secondary) | 0.10 | 1.10 | 1.093–1.113 | 0.14 | 1.16 | 1.140–1.169 | |
| Education (Tertiary) | 0.06 | 1.06 | 1.047–1.066 | <0.01 | 1.01 | 0.994–1.019 | |
| Marital status (single) | 0.12 | 1.13 | 1.124–1.138 | 0.01 | 1.00 | 0.992–1.010 | |
| Household (One-person) | 0.28 | 1.32 | 1.312–1.331 | 0.40 | 1.49 | 1.469–1.501 | |
| SO (sexual minority) | 0.13 | 1.14 | 1.131–1.153 | 0.06 | 1.06 | 1.042–1.072 | |
| ES (not employed) | 0.09 | 1.09 | 1.084–1.101 | 0.06 | 1.06 | 1.052–1.074 | |
| General anxiety (increased) | 2.15 | 8.57 | 8.519–8.618 | 2.19 | 8.89 | 8.819–8.964 | |
| Family relationships (worsened) | 0.99 | 2.70 | 2.677–2.723 | 1.00 | 2.72 | 2.689–2.754 | |
| Friendships (worsened) | 1.22 | 3.39 | 3.369–3.412 | 1.24 | 3.45 | 3.423–3.485 | |
| Alcohol use (increased) | 0.71 | 2.04 | 2.021–2.059 | 0.60 | 1.82 | 1.795–1.841 | |
| Tobacco use (increased) | 0.50 | 1.64 | 1.624–1.662 | 0.13 | 1.14 | 1.122–1.161 | |
| Income (decreased) | 0.36 | 1.43 | 1.425–1.444 | 0.40 | 1.50 | 1.483–1.510 | |
| Workload (increased) | 0.40 | 1.49 | 1.479–1.501 | 0.41 | 1.51 | 1.498–1.528 | |
| Symptoms (any) |  |  |  | -0.67 | 0.51 | 0.499–0.524 | |
| COVID (> 1 – 2 weeks) |  |  |  | 0.27 | 1.31 | 1.297–1.320 | |
| COVID (> 2 – 4 weeks) |  |  |  | 0.17 | 1.19 | 1.173–1.209 | |
| COVID (> 4 – 8 weeks) |  |  |  | 1.33 | 3.78 | 3.694–3.876 | |
| COVID (> 8 weeks) |  |  |  | 1.76 | 5.79 | 5.634–5.939 | |

*Notes*. *B* = logistic regression coefficient; OR = odds ratio; 95% CI = 95% confidence interval for the OR; SO = Sexual orientation; ES = Employment status. Predictors are listed with the respective comparison group in parentheses (e.g., Gender (Women) compares women to the reference group: men). All coefficients were statistically significant at *p* < .001, except for Area (Rural) in the extended model (*p* = .026), and Education (Tertiary) as well as Marital status (Single), which were not statistically significant (*p* > .05, respectively). All standard errors ranged between 0.003 and 0.013.
