# Supplementary material for: Predictors of Loneliness, Mental Wellbeing, and Stress During the COVID-19 Pandemic in Switzerland
Source: Int J Public Health. 2026 Feb 27;71:1609518. doi: 10.3389/ijph.2026.1609518 (PMC12982157; doi:10.3389/ijph.2026.1609518)
Supplement: Supplementary file 3 [file Table3.docx]

**Supplementary Table 3.**

*Binary Logistic Regression Predicting Increased Stress.*

| Predictor (comparison group) | Core Model | | | Extended Model | | |
| --- | --- | --- | --- | --- | --- | --- |
|  | *B* | OR | 95% CI | *B* | OR | 95% CI |
| Age (40 – 64) | -0,17 | 0,84 | 0.837–0.846 | -0,24 | 0,78 | 0.778–0.790 |
| Age (65+) | -0,60 | 0,55 | 0.544–0.554 | -0,52 | 0,59 | 0.584–0.601 |
| Gender (Women) | 0,39 | 1,47 | 1.465–1.479 | 0,40 | 1,50 | 1.489–1.508 |
| Nationality (Non-Swiss) | 0,02 | 1,02 | 1.010–1.025 | 0,05 | 1,05 | 1.039–1.061 |
| Migration (migration past) | 0,20 | 1,23 | 1.218–1.234 | 0,20 | 1,22 | 1.210–1.232 |
| Language region (French) | -0,03 | 0,97 | 0.965–0.976 | -0,03 | 0,97 | 0.967–0.981 |
| Language region (Italian) | 0,19 | 1,21 | 1.197–1.224 | 0,24 | 1,27 | 1.252–1.290 |
| Area (Intermediate) | -0,11 | 0,90 | 0.894–0.904 | -0,02 | 0,98 | 0.971–0.987 |
| Area (Rural) | -0,16 | 0,85 | 0.848–0.859 | -0,14 | 0,87 | 0.859–0.875 |
| Education (Secondary) | -0,20 | 0,82 | 0.810–0.822 | -0,14 | 0,87 | 0.862–0.880 |
| Education (Tertiary) | -0,27 | 0,77 | 0.761–0.773 | -0,22 | 0,80 | 0.791–0.807 |
| Marital status (single) | 0,08 | 1,08 | 1.072–1.083 | 0,06 | 1,06 | 1.056–1.072 |
| Household (One-person) | 0,10 | 1,11 | 1.102–1.116 | 0,04 | 1,05 | 1.035–1.055 |
| SO (sexual minority) | 0,23 | 1,26 | 1.249–1.270 | 0,34 | 1,41 | 1.391–1.424 |
| ES (not employed) | -0,30 | 0,74 | 0.738–0.748 | -0,36 | 0,70 | 0.693–0.706 |
| General anxiety (increased) | 2,43 | 11,31 | 11.250–11.376 | 2,40 | 10,98 | 10.896–11.068 |
| Family relationships (worsened) | 0,72 | 2,06 | 2.040–2.075 | 0,68 | 1,97 | 1.942–1.990 |
| Friendships (worsened) | 0,72 | 2,06 | 2.050–2.074 | 0,76 | 2,15 | 2.127–2.163 |
| Alcohol use (increased) | 0,59 | 1,80 | 1.784–1.815 | 0,62 | 1,86 | 1.838–1.881 |
| Tobacco use (increased) | 0,76 | 2,14 | 2.113–2.157 | 0,76 | 2,13 | 2.099–2.160 |
| Income (decreased) | 0,38 | 1,46 | 1.452–1.469 | 0,33 | 1,39 | 1.376–1.399 |
| Workload (increased) | 0,99 | 2,70 | 2.685–2.717 | 1,06 | 2,87 | 2.851–2.897 |
| Symptoms (any) |  |  |  | -0,35 | 0,71 | 0.695–0.720 |
| COVID (> 1 – 2 weeks) |  |  |  | 0,30 | 1,35 | 1.340–1.360 |
| COVID (> 2 – 4 weeks) |  |  |  | 0,48 | 1,62 | 1.599–1.640 |
| COVID (> 4 – 8 weeks) |  |  |  | 0,36 | 1,44 | 1.400–1.470 |
| COVID (> 8 weeks) |  |  |  | 1,05 | 2,85 | 2.772–2.921 |

*Notes*. *B* = logistic regression coefficient; OR = odds ratio; 95% CI = 95% confidence interval for the OR; SO = Sexual orientation; ES = Employment status. Predictors are listed with the respective comparison group in parentheses (e.g., Gender (Women) compares women to the reference group: men). All coefficients were statistically significant at *p* < .001. All standard errors ranged between 0.002 and 0.013.
